# Communication Delay Outlier Detection and Compensation for Teleoperation Using Stochastic State Estimation

**DOI:** 10.3390/s24041241

**Published:** 2024-02-15

**Authors:** Eugene Kim, Myeonghwan Hwang, Taeyoon Lim, Chanyeong Jeong, Seungha Yoon, Hyunrok Cha

**Affiliations:** Korea Institute of Industrial Technology, Gwangju 61012, Republic of Korea; egkim@kitech.re.kr (E.K.); han9215@kitech.re.kr (M.H.); limty226@kitech.re.kr (T.L.); hana2036@kitech.re.kr (C.J.); yoonsh@kitech.re.kr (S.Y.)

**Keywords:** teleoperation, autonomous vehicle, communication delay, outlier detection

## Abstract

There have been numerous studies attempting to overcome the limitations of current autonomous driving technologies. However, there is no doubt that it is challenging to promise integrity of safety regarding urban driving scenarios and dynamic driving environments. Among the reported countermeasures to supplement the uncertain behavior of autonomous vehicles, teleoperation of the vehicle has been introduced to deal with the disengagement of autonomous driving. However, teleoperation can lead the vehicle to unforeseen and hazardous situations from the viewpoint of wireless communication stability. In particular, communication delay outliers that severely deviate from the passive communication delay should be highlighted because they could hamper the cognition of the circumstances monitored by the teleoperator, or the control signal could be contaminated regardless of the teleoperator’s intention. In this study, communication delay outliers were detected and classified based on the stochastic approach (passive delays and outliers were estimated as 98.67% and 1.33%, respectively). Results indicate that communication delay outliers can be automatically detected, independently of the real-time quality of wireless communication stability. Moreover, the proposed framework demonstrates resilience against outliers, thereby mitigating potential performance degradation.

## 1. Introduction

Arbitrating control authority between a human driver and an automation system is regarded as a crucial function until fully automated control is successfully commercialized [1]. Due to erroneous driving behavior caused by the automatic control system of so-called self-driving vehicles, human drivers still play an important role in conducting maneuvering tasks. Because fully automated driving is focused on removing the human driver from the control loop, it liberates the human driver from tedious maneuvering but, consequently, leads to a loss in driving flexibility. Despite the dramatic development of artificial intelligence and the powerful computational performance of control units, there is not much doubt that it is challenging to completely remove the human driver from the conventional control loop [2,3].

Over the past few years, many researchers have shown an interest in the teleoperation of road vehicles to counter the uncertainty of a fully automated control system [4]. The typical teleoperation case scenario includes emergency takeover in urban driving and extra services like valet parking. However, it is also undeniable that human drivers also have uncertainty in terms of cognition of circumstances, bad driving performance caused by accumulated fatigue, and unquantifiable mental status properties which account for a large number of traffic accidents [5]. Therefore, it is important to arbitrate the control authority between the vehicle and the teleoperator, especially when wireless communication delay is concurrent. The main reason for the noticeable asynchrony between the teleoperator’s control and the actual mobility of the vehicle originates from a large number of network communication delays [6]. This degradation of mobility performance can be dramatic when exposed to environmental conditions such as tunnels with no wireless network coverage, mountainous regions, and malicious attacks such as jamming. Hence, when introducing a teleoperation scheme for an actual road vehicle, communication delays should be continuously monitored and evaluated based on the required safety integrity level in order to avoid hazardous situations.

The works of literature regarding teleoperation can be largely classified into human-in-the-loop studies [7,8,9,10] and driving performance evaluations [11,12,13]. In fact, numerous articles have already shown that task completion time increases depending on latency, regardless of the variety of target tasks, e.g., pin transfer [14], energy dissection [15], needle task [15], surgery [16], block transfer [17], and so on. In particular, a predictive display approach was introduced to forecast vision information to improve drivers’ cognition during teleoperation [18,19,20]. What should be noted is that some of the articles report that forecasting camera information considering communication delay can aid in improving human performance and workload while teleoperating a remote target [21,22]. Thus, methods like the predictive display can reduce the asynchrony between the perception of the teleoperator and the target vehicle’s response approach, which requires adaptive state estimation of communication delay.

There are two main approaches to handling sample outliers. One is a compensation strategy and the other is a detect-to-reject strategy. First, as the name suggests, the compensation method sets the allowable range of the sample value using a method appropriate for the physical parameter [23,24,25]. In general, when using multivariate variables, there is a method for setting a normalized allowable area based on the Mahalanobis distance, and it is common to set a threshold appropriate for other safety standards or purposes [24]. Second, in the case of the detect-to-reject strategy, the outlier detection part of the compensation method is the same, but the stability of the system is improved by removing samples [26,27]. However, none the proposed methods addresses the fact that they cannot directly handle delay samples because communication delay is considered as additional information related to physical parameters. In contrast, this study can be used for the detection of communication delay, and it is up to the filter designer’s discretion how to treat the physical parameters associated with it.

There has been great discussion about performance evaluation regarding teleoperation. For instance, bilateral teleoperation systems, at their core, are inherently feedback-driven systems, and they lead to a critical factor in the equation of transmission delay. Without any proper considerations, the communication delay can, at best, compromise the effectiveness of the closed-loop control and, at worst, jeopardize the stability of the entire bilaterally controlled teleoperation system. In order to maintain the robustness of the system, the predictive approach for bilateral teleoperation was designed, assuming that the communication delay is measured and bonded with constraints [28]. However, it is essential to focus on whether the communication outlier exists and, moreover, whether the robustness of the system can be guaranteed if a communication delay coexists. For this reason, there is a possibility that the robustness of many reported evaluation methods may not be preserved if outliers of communication delay are not considered in common scenarios, such as teleoperation via shared impedance control [29], delayed bilateral control [30], evaluation of display methods [18], and so on. In addition, several studies have proved that compensation strategies are effective in reducing the effect of large communication delays on vehicle mobility [6,31,32,33]. Again, what seems to be lacking is the considerations regarding communication outlier detection and the implementation of countermeasures. Therefore this research was undertaken regarding communication delay estimation and evaluations and further detecting communication delay outliers.

### Contributions

This study’s topic is communication delay outlier detection for teleoperation of a remote vehicle using stochastic state estimation, and the contributions of this study are as follows:

- The primary aim of this study was to assess outliers in communication delays during real-time teleoperation accurately. Since communication delays are influenced by various environmental factors such as signal quality, channel conditions, buffer status, and network load, distinguishing erroneous outliers in real time poses a significant challenge. Traditional outlier detection methods typically rely on predetermined rules based on empirical samples. However, our proposed approach employs safety-oriented criteria utilizing a coverage interval to evaluate acceptable delay thresholds dynamically. Consequently, we introduced stochastic criteria for promptly identifying communication delay outliers. Particularly, we demonstrated the efficacy of our outlier detection algorithm by showcasing enhanced performance through compensatory actions for detected outliers when employing a predictor-based framework approach;
- Moreover, this study relied on actual communication delay measurements achieved through GPS time synchronization. Unlike traditional methods that measure round-trip delay, we focused on estimating one-way communication delay outliers. To accomplish this, we synchronized the time between the vehicle and the control center using GPS and compared the synchronized times to gauge one-way communication delay. This approach offers practicality for various wireless communication applications and furnishes guidance on measuring one-way communication delay, applicable not only to vehicles but also to remote control scenarios involving robots, agricultural machinery, and embedded systems;
- The findings indicate that the proposed approach is versatile and suitable not only for stable communication environments but also for dynamic conditions. Particularly, it effectively handles unpredictable communication delays, such as those encountered when transmitting between continents or traversing shielded spaces where electromagnetic waves face obstacles. An important feature of the proposed method is its ability to detect communication anomalies based on emerging trends without prior knowledge of the communication delays in the specific area. Consequently, it offers the advantage of applicability in dynamic environments, with or without prior local knowledge, making it valuable for remote work scenarios.

## 2. Methodology

This section presents various schemes related to communication delay measurement. Firstly, the network time protocol (NTP) is elucidated from the perspective of time synchronization. Secondly, a state estimation predictor is introduced for classifying communication delays.

### 2.1. Network Time Protocol (NTP)

**Figure 1:**
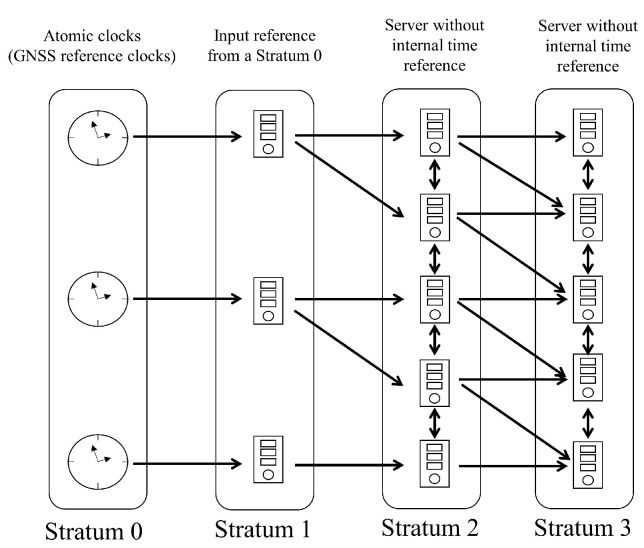
Schematic diagram showing the layer of the network time protocol where distributed layered systems using independent time sources are synchronized hierarchically (*n*th stratum is connected to n+1th stratum).

Let there be a layered system using independent time sources. Then, each system can be organized hierarchically, which is termed as a stratum. Each stratum consists of *n* synchronized stratum servers, and the number *n* depicts the distance from the reference clock system, which is stratum 0. Note that the number of the stratum may not always indicate the quality of the inner clock’s precision or accuracy. Then, the whole structure of the layered system is as shown in Figure 1.

**Figure 2:**
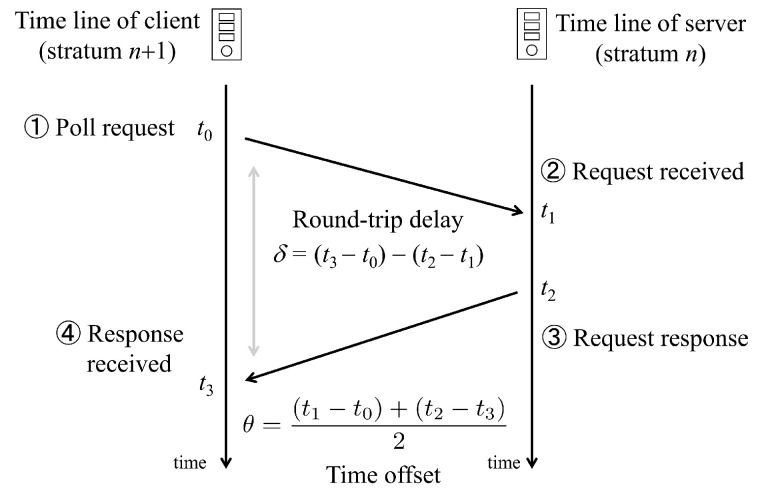
Diagram showing the procedure of the clock synchronization algorithm, where δ is the round-trip delay, θ is the offset, $t_{0}$ is the time when a poll is requested from the client, $t_{1}$ is the time the poll request is received from the server, $t_{2}$ is the time of response from the server, and, finally, $t_{3}$ is the time the response is received by the client.

In detail, a regular network time protocol (NTP) can be established using the above-mentioned layered system, and the client calculates a communication time offset and a round-trip delay as follows [34]:

(1) $$\delta =\left(t_{3}-t_{0}\right)-\left(t_{2}-t_{1}\right),$$

where δ is the round-trip delay, $t_{0}$ is the time when a poll is requested from the client, $t_{1}$ is the time when the poll request is received from the server, $t_{2}$ is the time of the response from the server, and, finally, $t_{3}$ is the time the response is received by the client as follows:

As shown in Figure 2, clock synchronization can be performed by computing the offset between the server and the client.

(2) $$\theta =\frac{\left(t_{1}-t_{0}\right)+\left(t_{2}-t_{3}\right)}{2},$$

where θ is the clock offset. It should be noted that the time offset can be a positive value or a negative value depending on the instance timeline information of the client and the server. Next, in order to derive the amount of the offset in real time, a request packet can be utilized, such as

(3) $$t_{0}=t_{1}-\theta -\delta /2,$$

for the case of the response packet, and

(4) $$t_{3}=t_{2}-\theta +\delta /2,$$

and, finally, the θ can be solved. However, it is important to remove the outliers of the estimated θ and δ to synchronize the inner clocks for the layered system.

The above-mentioned NTP can be utilized based on a global positioning system (GPS) or a global navigation satellite system (GNSS) when applied to individual vehicles. After decoding, NMEA (National Marines Electronics Association)-0183 sentences, which are encoded packets received from multiple satellites at the time when the packets are sent, can be known [35]. However, a fundamental problem of using NMEA sentences is that there is no firm timing protocol for when to initiate packet sending after a new second starts. Therefore, it is important to know that, if the client receives an NMEA packet, it does not have any evidence of whether it has been sent at the start of the new second or the end of the new second. For this issue, 1 pulse per second (PPS) can be useful for enhancing the NMEA to have better accuracy. The 1-PPS signal is a type of pulse signal that indicates the start of the new seconds and is considered to have less than 40 ns average precision. Therefore, by combining the NMEA sentence timestamps and the 1-PPS signal, the system can have better accuracy and better precision [36].

**Figure 3:**
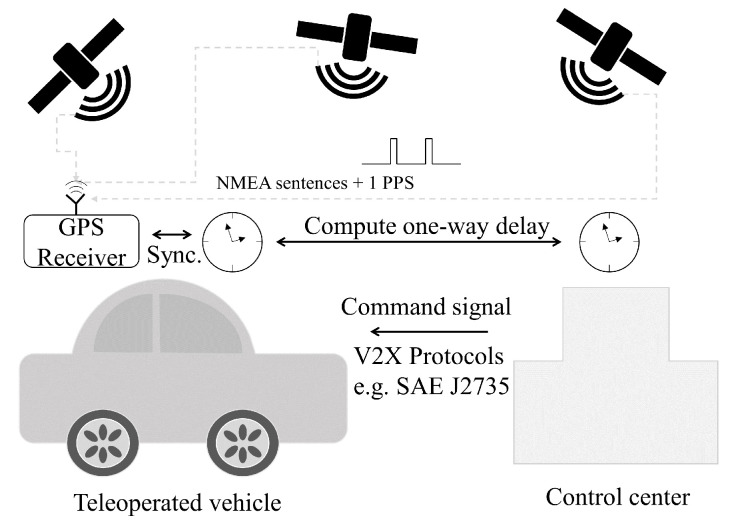
Example of one-way communication delay measurement using NTP server based on the GPS timestamp synchronization [38].

Figure 3 shows the one-way communication delay measurement using the NTP server shown in the above. After both the teleoperated vehicle and the control center have synchronized clock systems, by sending the sent time with the control command from the control center, a vehicle controller inside of the teleoperated vehicle is able to compute the one-way communication delay. In this scenario, numerous approaches to the communication method can be used such as (but not limited to) 5G/LTE and DSRC [37] with appropriate protocols, e.g., SAE J2735 [38]. As for the common failure scenarios for using such wireless communication methods, the performance of the connectivity can be degraded due to systematic failures, low sensitivity, and even weather conditions [39]. Therefore, careful monitoring of the instant state of communication is required, especially when implementing teleoperation of the vehicle.

### 2.2. State Estimation of Communication Delay

Let $T_{s}$ be the synchronized time of the slave which is the timestamp of the remote vehicle and $T_{m}$ be the time of the master which is the timestamp of the control center. Then, the one-way communication delay $T_{c}$ can be computed with synchronized time information as follows:

(5) $$T_{c}=T_{s}-T_{m}.$$

**Figure 4:**
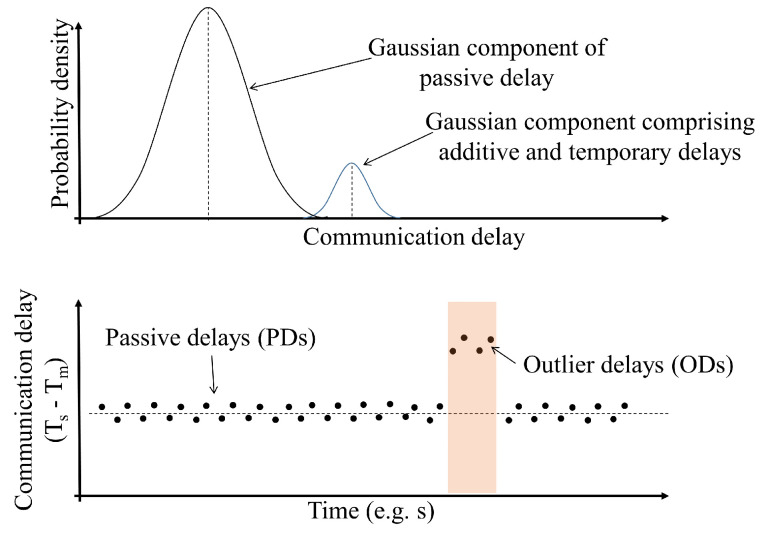
Classification of communication delays based on the measured one-way communication delay $T_{c}$, where $T_{s}$ is the synchronized time of the time slave node, and $T_{m}$ is the synchronized time of the master node.

In this study, as shown in Figure 4, we categorize the communication delay into two types of modes which are passive communication delay (PD) and outlier communication delay (OD). In order to distinguish ODs from PDs, a Gaussian mixture model can be used to express the distributions using linear multiple Gaussians as follows:

(6) $$p\left(T_{c}\right)=\sum_{k=1}^{K}\pi_{k}N\left(T_{c}|\mu_{k},\Sigma_{k}\right),$$

where *k* is the latent Gaussian variable, *K* is the number of Gaussian components, $T_{c}$ is the vector of the set of one-way communication delay samples, $\pi_{k}$ is the mixture coefficient of the *k*th Gaussian, N refers to the Gaussian, $\mu_{k}$ is the means of the latent Gaussian components, and $\Sigma_{k}$ is the variations of the latent Gaussian components.

In order to derive (6), a discrete random variable *z* is introduced, which, having *K*-dimension $z_{k}\in \left\{0,1\right\}$, can be defined as a one-hot encoded variable consisting of the specific element as 1 and the other as 0, which is $\sum_{k}z_{k}=1$. The variable *z* can have a different *K* state depending on whether the specific element is 0 or not. The joint distribution p(x,z) can be defined with marginal distribution p(z) and conditional distribution p(x|z) as follows:

(7) $$p\left(z_{k}=1\right)=\pi_{k},$$

where $\pi_{k}$ is a parameter which should meet the following condition:

(8) $$\begin{array}{c} 0\leq \pi_{k}\leq 1 \end{array}$$

(9) $$\begin{array}{c} \sum_{k=1}^{K}\pi_{k}=1. \end{array}$$

Finally, the upper distribution can be written as:

(10) $$p\left(z\right)=\prod_{k=1}^{K}\pi_{k}^{z_{k}}.$$

Similarly, when a specific z is given, the conditional distribution regarding the vector of one-way communication delay $T_{c}$ can be written as Gaussian distribution as follows:

(11) $$p\left(T_{c}|z_{k}=1\right)=N\left(T_{c}|\mu_{k},\Sigma_{k}\right),$$

which can be expressed also as:

(12) $$p\left(T_{c}|z\right)=\prod_{k=1}^{K}N{\left(T_{c}|\mu_{k},\Sigma_{k}\right)}^{z_{k}}.$$

Since the joint distribution is given as $p\left(z\right)p\left(T_{c}|z\right)$, marginal distribution can be derived as follows:

(13) $$p\left(T_{c}\right)=\sum_{z}p\left(z\right)p\left(T_{c}|z\right)=\sum_{k=1}^{K}\pi_{k}N\left(T_{c}|\mu_{k},\Sigma_{k}\right).$$

On the other hand, a likelihood function of the derived joint distribution can be written as:

(14) $$L\left(\theta ;T_{c|1},\ldots ,T_{c|N}\right)=P\left(T_{c|1},\ldots ,T_{c|N};\theta \right)=\prod_{i=1}^{K}N\left(T_{c|i}|\mu ,\sigma^{2}\right),$$

where θ=(μ,σ) is the parameter of the Gaussian distributions, $T_{c|}i$ is the *i*th communication delay sample, *N* is the number of samples, μ is mean of samples, and σ is the standard deviation of the samples. Next, the maximum value of the logarithm of the likelihood function does not change and can be written as:

(15) $$\begin{array}{c} \log L\left(\theta ;T_{c|1},\ldots ,T_{c|N}\right)=\sum_{i=1}^{N}N\left(T_{c|i}|\mu ,\sigma \right)\\ =-\frac{1}{2}\sum_{i=1}^{K}\frac{{\left(T_{c|i}-\mu \right)}^{2}}{\sigma^{2}}-\frac{N}{2}\log\left(2\pi \sigma^{2}\right). \end{array}$$

In other words, since we assume the samples were extracted independently, the joint distribution can be rewritten as the equivalent sample set as follows:

(16) $$\log p\left(T_{c}|\pi ,\mu ,\Sigma \right)=\sum_{i=1}^{N}\sum_{k=1}^{K}\pi_{k}N\left(T_{c|i}|\mu_{i},\sigma_{i}\right).$$

By using the expectation maximization (EM) method, (15) can be used to estimate the *K* Gaussian distributions as follows:

(17) $$P\left(\pi_{k}|T_{c},\lambda \right)=\frac{\pi_{k}N\left(T_{c|i}|\mu_{k},\Sigma_{k}\right)}{\sum_{k=1}^{K}\pi_{i}N\left(T_{c|i}|\mu_{i},\Sigma_{i}\right)},$$

where λ is a parameter of Gaussians ($\lambda =\{\pi_{k},\mu_{k},\Sigma_{k}\}$), and $\pi_{k}$ is the weight of the *k*th Gaussian among the *K* Gaussians, which can be written as:

(18) $${\hat{\pi }}_{k}=\frac{1}{N}\sum_{i=1}^{N}P\left(k|T_{c|i},\lambda \right),$$

where ${\hat{\pi }}_{k}$ is the estimated weight of the *k*th Gaussian. Finally, the means and variances can be estimated as:

(19) $$\begin{array}{cc} {\hat{\mu }}_{k} & =\frac{\sum_{i=1}^{N}P\left(k|T_{c|i},\lambda \right)T_{c|i}}{\sum_{i=1}^{N}P\left(k|T_{c|i},\lambda \right)} \end{array}$$

(20) $$\begin{array}{cc} {\hat{\sigma }}_{k} & =\frac{\sum_{i=1}^{N}P\left(k|T_{c|i},\lambda \right)T_{c|i}^{2}}{\sum_{i=1}^{N}P\left(k|T_{c|i},\lambda \right)}-{\hat{\mu }}_{k}^{2}, \end{array}$$

where ${\hat{\mu }}_{k}$ and ${\hat{\sigma }}_{k}$ denote the expected mean and standard deviation of the *k*th Gaussian during *K* Gaussians.

**Figure 5:**
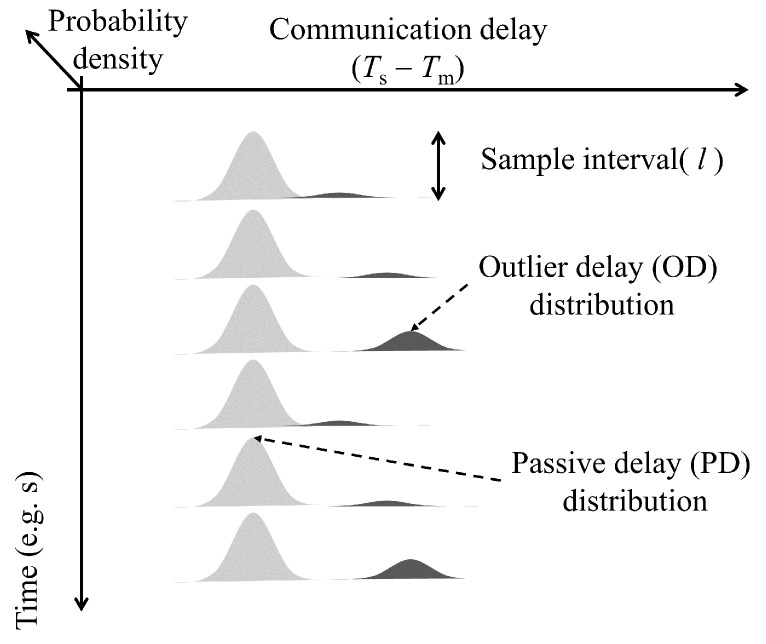
Proposed method to detect outlier delay (OD) using Gaussian mixture model.

Figure 5 shows a schematic diagram of the proposed method to distinguish ODs from PDs. Furthermore, it can be written as the following equation:

(21) $$P\left(\pi_{k}|T_{c|i-l|i-1},\lambda \right)=\frac{\pi_{k}N\left(T_{c|i-l|i-1}|\mu_{k},\Sigma_{k}\right)}{\sum_{k=1}^{K}\pi_{k}N\left(T_{c|i-l|i-1}|\mu_{k},\Sigma_{k}\right)},$$

where *l* is the sample interval. By using Equation (21), the communication delays can be estimated in real time to distinguish ODs and also can be used as a judgment criterion for the instant communication delay $T_{c|i}$.

### 2.3. Outlier Judging Metric

In order to distinguish the outlier dynamically, a stochastic outlier judging criterion is used. One of the sophisticated methods to detect outliers, the evaluation means used in the safety-related sensor system of the safety standard IEC-62998, is referenced [40]. The core idea of the coverage interval is to determine the threshold to validate that the measured samples are kept inside the boundary for a certain probability as follows [27]:

(22) $$C_{p}\leq 1-\frac{PFH_{u}}{d},$$

where $C_{p}$ is coverage probability, $PFH_{u}$ is the probability of failure in an hour, and *d* is the demand rate, which is how frequently the functionality of the system is required to operate. Finally, the judgment criterion for the ODs can be written as:

(23) $$P(\gamma \left(T_{c}\right)\geq \chi_{\alpha })=\alpha ,$$

where $\chi_{\alpha }$ is the predetermined α-quantile of the Chi-square distribution. In other words, α can be written as a significance level. In this framework, outliers will be detected based on the computed probability threshold that refers to α. In this approach, based on the safety integrity level (SIL) of 2, it is assumed that the target system which makes α should be higher than $1-2.5\times 10^{-7}$. $\chi_{\alpha }$ is therefore set as 26.62 [41]. In addition, γ denotes the Mahalanobis distance.

(24) $$\gamma \left(T_{c}\right)=M^{2}={\left(T_{c}-\mu \right)}^{T}{\left(\sigma \right)}^{-1}\left(T_{c}-\mu \right),$$

where *M* is the Mahalanobis distance criterion. If judging index $\gamma (T_{c})$ is larger than $\chi_{\alpha }$, the outlier is rejected because the safety criterion is violated. It should be noted that, according to the dimension of the measured physical parameters, the domain of the $\chi_{\alpha }$ will be changed.

### 2.4. Outlier Compensation Predictor-Based Framework for Teleoperated System

In this study, to show the possibility of integrating the proposed framework with other techniques or strategies for enhancing teleoperation performance, we used the predictor-based framework proposed by Zheng et al. to minimize the effect of communication delay with the teleoperator on the mobility of the teleoperated vehicle [6,31,32].

First, communication signals y(t) were defined to include steering δ. The authors added the throttle pedal value and brake value through y(t); however, since this study aimed to improve teleoperation performance through the detection of communication delay, only the steering angle value was used for simplicity. In addition, the authors divided the variables into $y_{1}\left(t\right)$ and $y_{2}\left(t\right)$ and considered them as command signals reaching the teleoperated vehicle and, conversely, sensor signals sent from the remote vehicle to the control center. However, in this study, only $y_{1}\left(t\right)$, the delay transmitted to the teleoperated vehicle, was considered.

Next, using the derivative of $\dot{y}\left(t\right)$, corresponding predicted results can be written as:

(25) $${\dot{\hat{y}}}_{i}\left(t\right)={\dot{y}}_{i}\left(t-T_{c|i}\left(t\right)\right)+\eta \left[y_{i}\left(t-T_{c|i}\left(t\right)\right)-{\hat{y}}_{i}\left(t-T_{c|i}\left(t\right)\right)\right],$$

where *i* is the number of communication channels, ${\hat{y}}_{i}\left(t\right)$ is the predictor space state, and ${\hat{y}}_{i}\left(t-T_{c|i}\left(t\right)\right)$ is the predicted communication signals of delayed time. In addition, η is the diagonal square matrix that has non-zero $\eta_{j}$(j=1,2,…,n) regarding the number of communication signal elements.

Despite numerous attempts by researchers to leverage the predictor-based framework to enhance stability and performance, as discussed in previous works [31], and endeavors to elucidate the optimal selection of the gain parameter η [32], the issue of ensuring consistent performance remains ambiguous. This ambiguity can particularly be pronounced in scenarios where communication outliers coexist, adding an additional layer of complexity to the assessment of system behavior. Therefore, (25) is modified to have two switching sequences:

(26) $${\dot{\hat{y}}}_{i}\left(t\right)=\left\{\begin{array}{cc} {\dot{y}}_{i}\left(t-T_{c|i}\left(t\right)\right)+\eta \left[y_{i}\left(t-T_{c|i}\left(t\right)\right)-{\hat{y}}_{i}\left(t-T_{c|i}\left(t\right)\right)\right] & \mathrm{if}\;P(\gamma \left(T_{c}\right)\leq \chi_{\alpha })\\ {\dot{\hat{y}}}_{i}\left(t-T_{c|i}\right) & \mathrm{if}\;P(\gamma \left(T_{c}\right)>\chi_{\alpha }). \end{array}\right.$$

The initial sequence aligns with the predictor-based framework as originally proposed by the authors. However, in the second scenario, when a communication delay is identified as an outlier, distinctive measures are employed to assess the system’s stability by using the derivative at time *t* expected from the previous state.

## 3. Experiment

In this section, experimental conditions are expressed, and general setups including apparatus are introduced. This study, however, does not contain any simulation approaches.

### 3.1. General Setups

**Figure 6:**
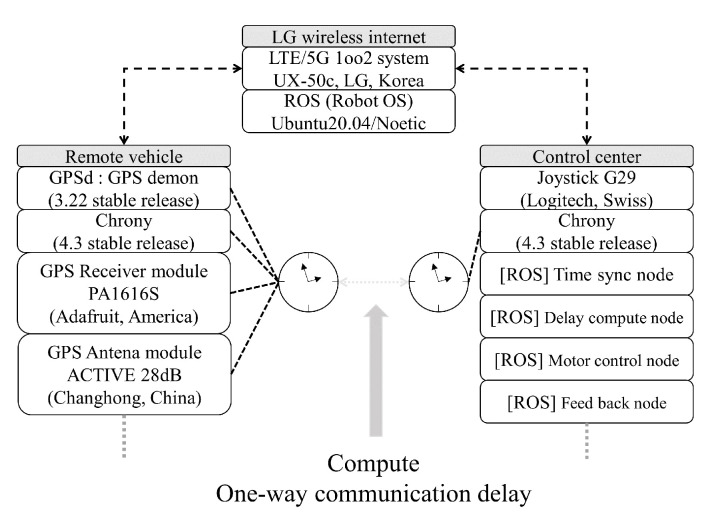
Experimental setups between the remote vehicle and the control center using wireless internet.

Figure 6 shows experimental setups including a wireless internet network system using LTE/5G networks. The system was constructed based on the ROS (Robot OS) system (Noetic, Ubuntu 20.04) for both the remote vehicle and the control center. More importantly, two distinct systems were set to use NTP synchronization using Chrony (4.3 stable). As for the GPS receiver, PA1616S (Adafruit, New York, NY, USA) and the GPS active module were used (Active 28 dB, Changhong, Mianyang, Sichuan, China). In order to receive the NMEA sentence samples and decode them, GPSd (3.22 stable release) was used. On the other hand, in order to teleoperate the remote vehicle, Joystick G29 (Logitech, Lausanne, Switerland) was used.

### 3.2. Communication Delay Measurement and Teleoperation Setups

**Figure 7:**
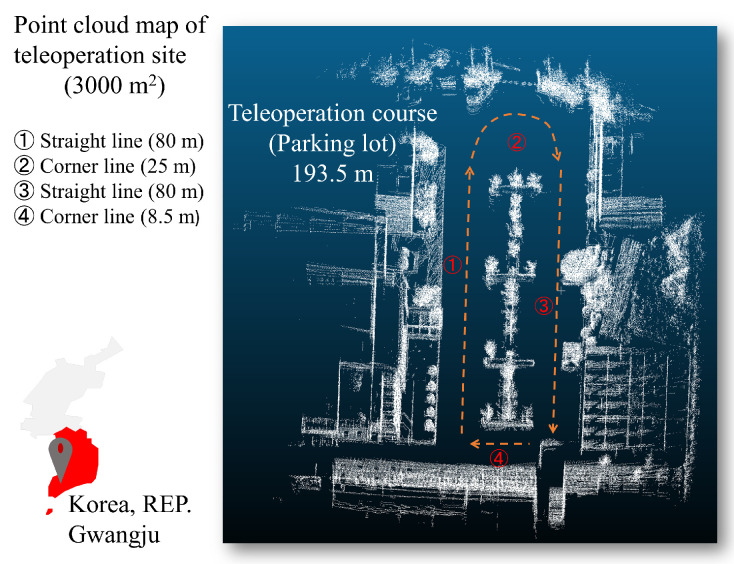
Teleoperation course of the test site (Seonam division, Gwangju, Republic of Korea). The total area of the site is 3000 m^2^, and the one-way length of the course is 193.5 m.

Figure 7 shows a teleoperation site for the communication delay measurement. The total area of the site is 3000 m^2^, and the one-way length of the course is 193.5 m. One-way communication delay samples were collected continuously from a remote vehicle for 30 min, and the collection interval was set to a maximum of 40 Hz. The vehicle was safely driven at a constant speed of 10 km/h or less considering stopping distance and delay time and traveled a total of 25 laps.

**Figure 8:**
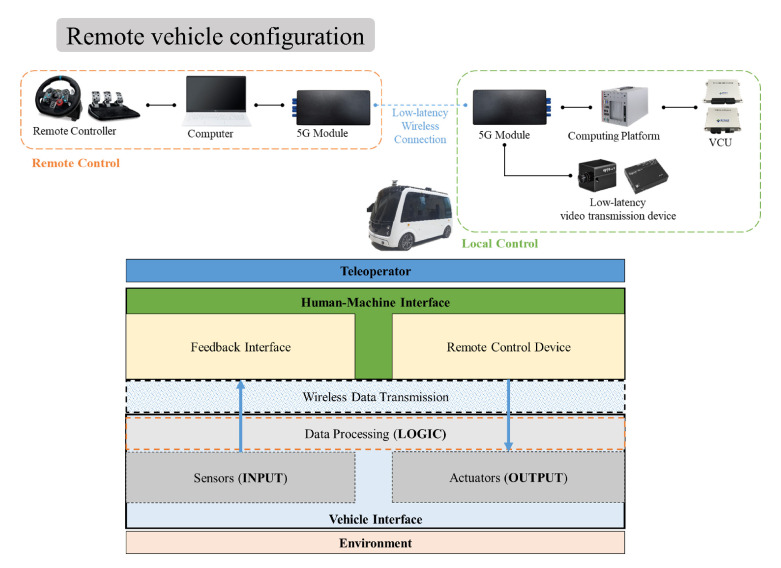
Vehicle setup of teleoperation using a CAN network to communicate with each low-level controller and camera communication.

**Figure 9:**
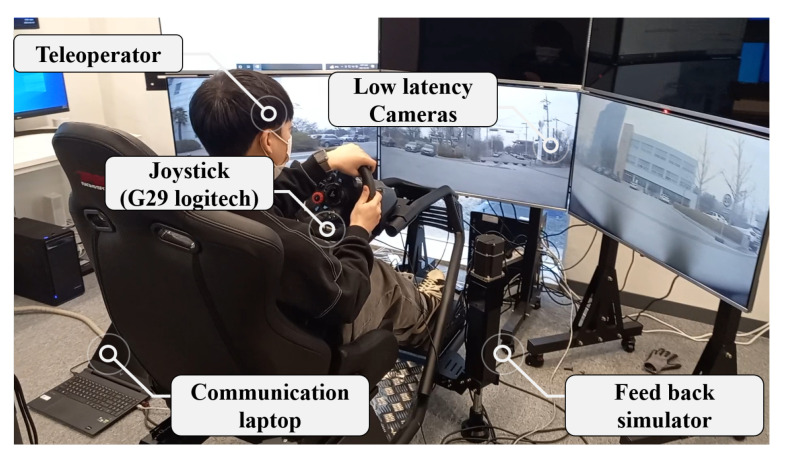
Experimental setup scene including feedback simulator.

Figure 8 and Figure 9 show the remote vehicle configuration and experimental setup scene. The remote vehicle uses a CAN (Controller Area Network) to connect lower-level controller terminals and has a communication speed of 250 kbps. In teleoperation, video transmission and reception performance are critical and conducted in an environment that can receive video at the control center using transmission and reception terminals that support video compression and low-latency camera terminals.

## 4. Results and Discussion

The computational complexity of the proposed model can be expressed in terms of the number of components (K), the dimensionality of the data (D), the number of iterations required for convergence, and the specific operations involved in the E step and M step of the EM algorithm. The overall complexity is considered to be on the order of O(K× D×N×I), where N is the number of data points, and I is the number of iterations. In addition, in most cases, parsing NMEA sentences is a relatively low-complexity task. The straightforward comma-separated format simplifies the parsing process, and many programming languages provide built-in functions or libraries to facilitate this task. The overall time complexity is often linear, O(N), where N is the length of the NMEA sentence.

### 4.1. Overall Communication Delay Analysis

**Figure 10:**
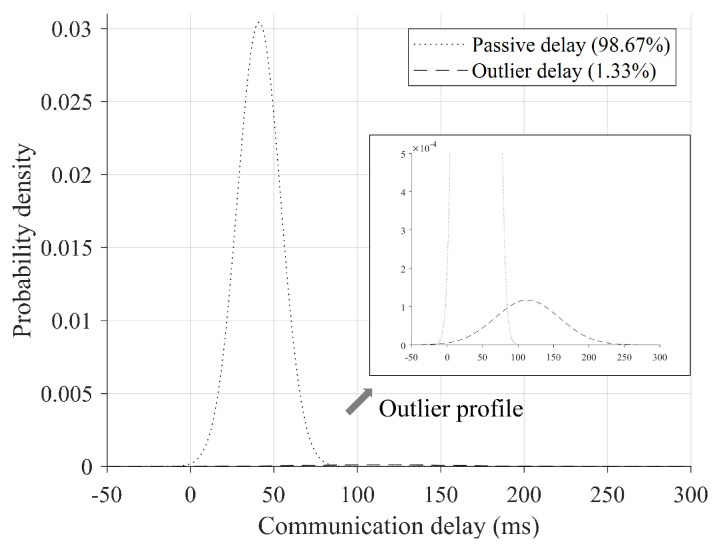
Result of probability density estimation for the overall measured communication delay $T_{c}$ samples.

Figure 10 shows the result of probability density estimation for the overall measured communication delay $T_{c}$ samples. The parameter *K* was set to 2 to distinguish the PDs from ODs, and weights of the PDs and ODs were estimated as 98.67% and 1.33%, respectively. As expected, the result of the probability density estimation for the overall measured communication samples was divided into two distinct Gaussian mixture models (Figure 10). The first Gaussian component, including passive communication delay, dominated 98.67%, and only 1.33% of the total communication delay was considered outlier delay (Figure 10). On the other hand, the second Gaussian component, including outlier delay, had a maximum outlier delay of 323 ms with an average of 113 ms (Figure 10). From the perspective of teleoperation, the smaller the size of the communication delay, the better, which can better guarantee real time; but, on the other hand, it is considered better to compensate as much as possible for delays of 100 ms or more, which can be felt by the human operators.

Meanwhile, many previous studies have evaluated communication delays based on round-trip time (RTT), and their characteristics differ due to reasons such as region and application system setup. In general, looking at previous studies, RTT shows various communication delay results ranging from 0.1 s to 1 s and appears to vary depending on the communication distance and type of network used [42,43]. Since this is about twice the difference from the one-way communication delay measured in this study, the actual one-way delay can be interpreted as having a range from 0.0 5 s to 0.5 s. In the case of 3G communication, it was found that there was an average RTT of 0.121 s and a maximum of 1 s or more, and, via a satellite network, there was a communication delay of about 0.75 s [42]. In addition, there was actually a study that measured one-way communication delay like this study, and it was found to have an uplink speed of 0.33 s and a video downlink speed of 0.55 s at which control commands are transmitted [43]. In other words, the communication delay results obtained in this study show relatively low communication speeds, suggesting that they are strongly dependent on the type of remote control application and the characteristics of the network used.

### 4.2. Weight of Gaussian Components

**Figure 11:**
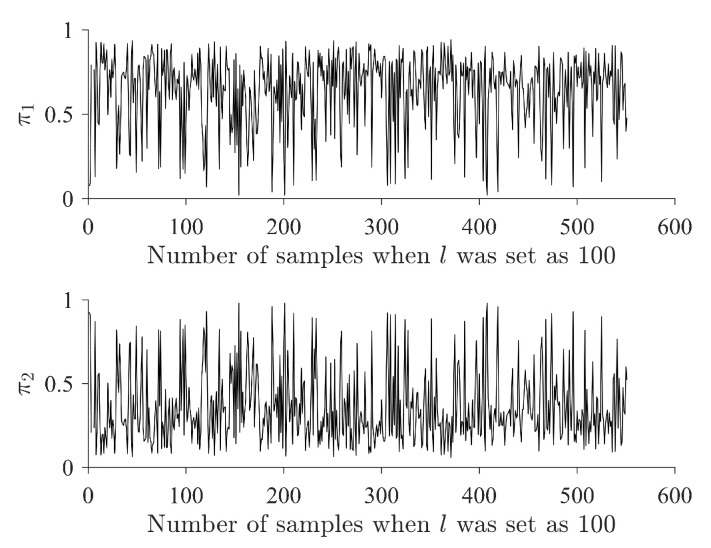
Weight analysis of Gaussian components when the sample interval *l* is set as 100.

Figure 11 shows the weight of the Gaussian components when the sample interval *l* was set as 100 (2.5 s of the time interval domain). In addition, from the weight of the estimated Gaussian components, the overall weight of the passive Gaussian component was greater than that of the second Gaussian component (${\bar{\pi }}_{1}$ > ${\bar{\pi }}_{2}$, Figure 11).

### 4.3. Outlier Classification Analysis

**Figure 12:**
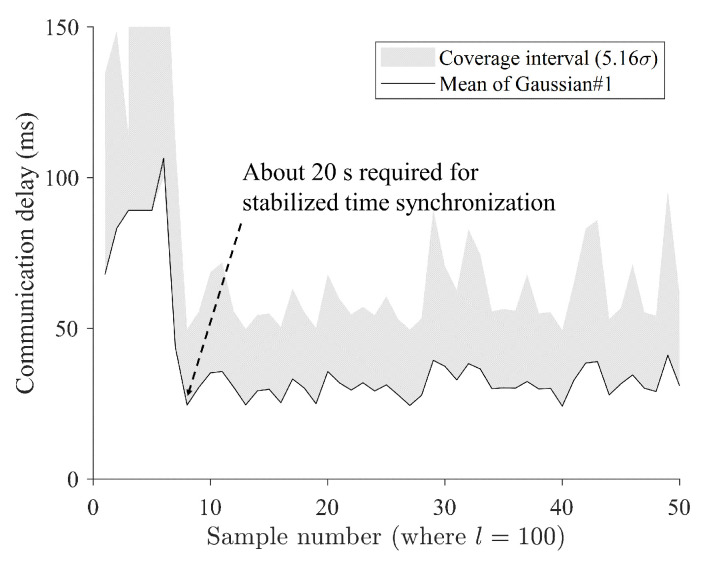
Required time analysis for stabilized time synchronization when *l* is set as 100.

Figure 12 shows a required time for stabilized time synchronization when *l* was set as 100. It was estimated that about 20 s was required to achieve stabilized synchronization. One thing to note when using the method proposed in this study is that there is a certain amount of waiting time for synchronization. The communication delay was confirmed to have a round-trip delay of 52 ms when conducted with a general ping test, which is about twice the size of the average communication delay of 29.3 ms measured in this experiment (Figure 12).

**Figure 13:**
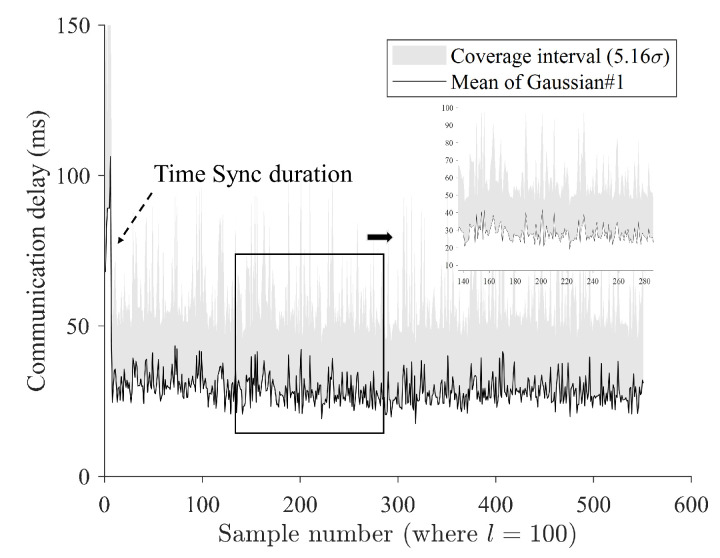
Estimated instant coverage interval when *l* is set as 100, showing that the overall mean value of the estimated first Gaussian is 29.36 ms.

Figure 13 shows an estimated instant coverage interval when *l* was set as 100. Since the method proposed in this study uses time synchronization using GPS, a certain amount of time is required to achieve time synchronization, and, in the experiment, stable communication time comparison was possible after about 20 s (Figure 13). Meanwhile, among the systems used in the experiment, the NTP-based time synchronization method using Chrony can set various offsets and synchronization cycles, so faster synchronization times can be expected by adjusting the settings based on the measurement environment.

**Figure 14:**
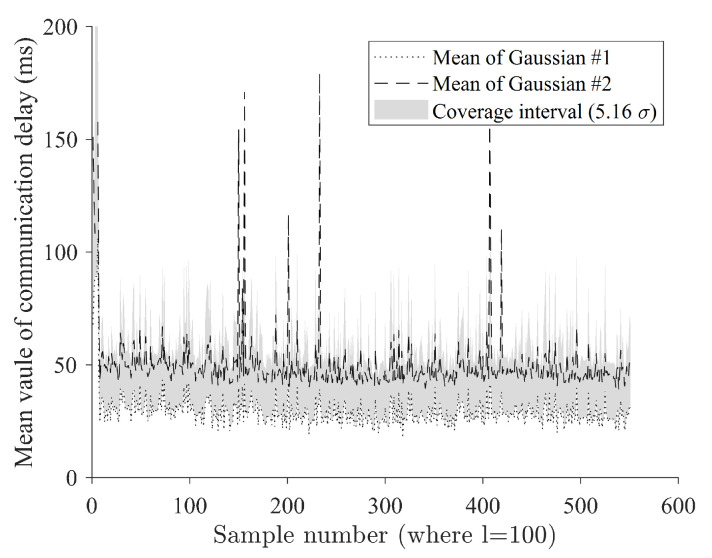
Comparison of instant coverage interval when *l* is set as 100 and estimated mean values of each Gaussian component.

Figure 14 shows the comparison of instant coverage interval and estimated mean values of each Gaussian component. Communication delay occurs as a complex condition and cannot escape the fundamental limitations of wireless communication. Regardless of the quality of wireless communication itself, the quality may deteriorate due to the momentary influence of the branch communication network. This is not only difficult to predict, but there is little basis for modeling it. Therefore, in this study, a dynamic judgment criterion using a probability-based coverage interval was used to adaptively design an instantaneous communication delay outlier (23). The area with a coverage interval of 5.16σ from the estimated average value of the first Gaussian was assumed to be an acceptable communication delay zone, and samples outside that area were defined as outliers (Figure 14).

**Figure 15:**
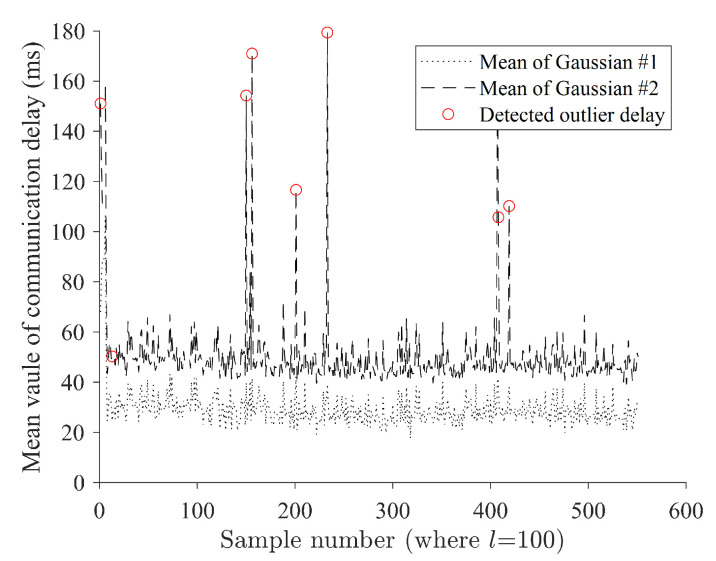
Result of detected outlier based on the estimated communication delay outlier.

Figure 15 shows the result of the detected outlier based on the estimated communication delay outlier. Note that the detected red circles denote the detected erroneous mean value of the second Gaussian component, which is the latent moment of the communication delay outlier, which is dominant at that instant. As a result, it was possible to capture moments that may include communication delay outliers, and the resolution was determined depending on the sample interval *l* set in advance (Figure 15). One thing to note is that the moments captured here are sections where outliers exist based on the sample interval, and the entire sample is not treated as a communication outlier. Therefore, it is necessary to design the sample interval to suit the needs, and, if the sample interval is overestimated or less discrete than necessary, a decrease in performance is expected.

**Figure 16:**
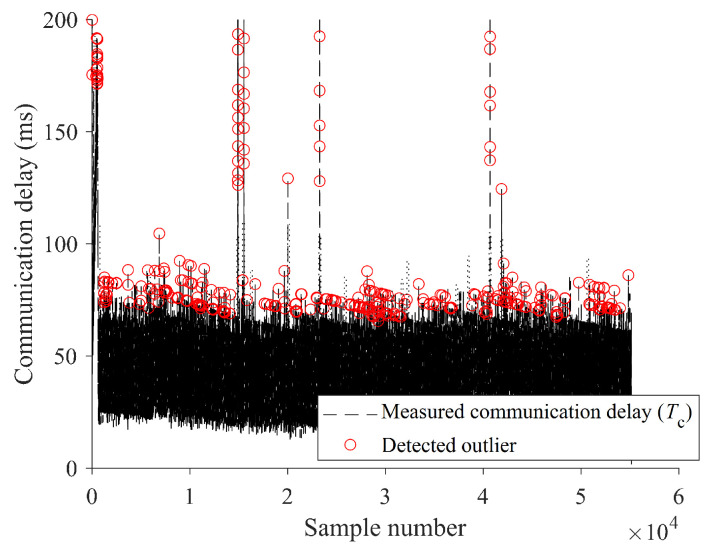
Total communication delay analysis based on the estimated coverage interval.

Figure 16 shows the total communication delay analysis based on the estimated coverage interval where the red markers represent the detected outlier delay samples among the total measured $T_{c}$. Among the total measured communication delay samples $T_{c}$, the communication delay outlier was distinguished (Figure 16). The communication delay outlier detected in this way is greatly affected by the coverage interval, and the sensitivity can be adjusted by adjusting the α value (23). In addition, the size of *l* described above must also be set in a way that meets the purpose of use, and tuning is required at the validation stage. Meanwhile, the measured communication delay value includes regional limitations, and, depending on the experimental environment, it includes even more communication delay in a wireless communication environment that crosses countries [6]. Since this study only presents a method for probabilistically determining communication delay outliers, it may not be useful if the overall communication signal is polluted by systematic abnormalities. Therefore, it is recommended to use the proposed method considering these constraints, and the failure mode according to system addition and application must be verified afterward.

### 4.4. Teleoperation Command Signal Analysis

**Figure 17:**
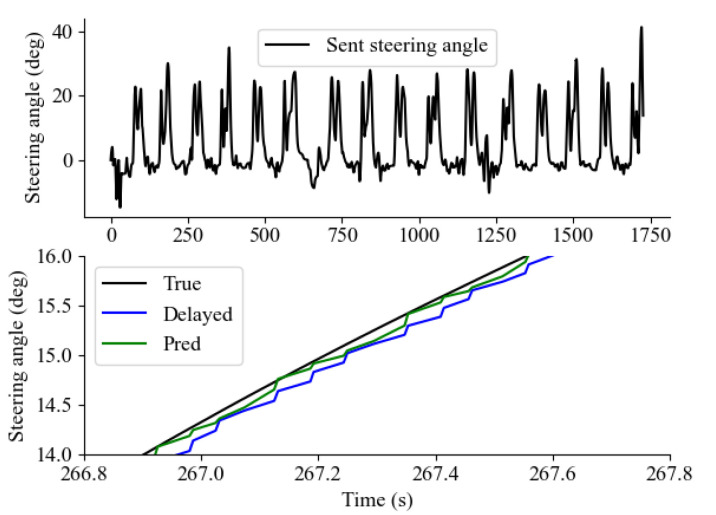
Overall teleoperation signal of sent steering angle comparing non-delayed signal, delayed signal, and predicted signal.

Figure 17 presents a comprehensive depiction of the teleoperation signal concerning the steering angle. Notably, given the prescribed course of the test site, which entails a consistent right rotation direction, a discernible bias towards positive values is evident in the overall distribution of steering angles. Additionally, the application of the predictor-based approach (denoted as ’Pred’) is noteworthy, as it appears to effectively alleviate the impact of signal delay on the received steering angle. It should be noted that the parameter λ was tuned using $0.3\lambda_{\max}$, where $\lambda_{\max}\left(T_{c}\left(t\right)\right)=3/\left(2{\bar{T}}_{c}\left(t\right)\right)$ [6].

**Figure 18:**
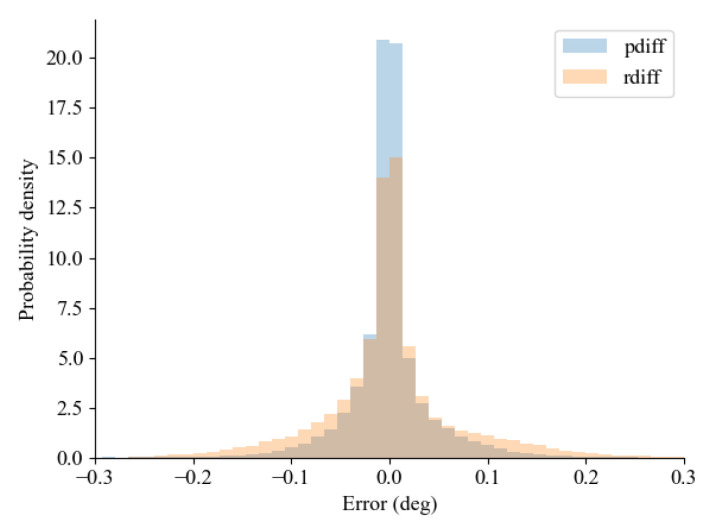
Comparison of error between the non-delayed signal and predictor-based approach with the delayed signal, where rdiff is a comparison of the delayed signal, and pdiff is a comparison of the predictor-based approach.

The probability density histogram of the estimated error between the non-delayed signal and the predictor-based approach with a delayed signal is illustrated in Figure 18. It is imperative to note that, due to disparate timestamps between the non-delayed signal and the delayed signals, a linear interpolation was employed for both delayed signals to facilitate the estimation of errors vis-à-vis the non-delayed signal. From the analysis, it may look like the error deviation of the delayed signal surpasses that of the predictor-based approach; however, the result of the computed standard deviations rather shows that the predictor-based approach (std.Predictor: 3.544) was higher than the delayed signal (std.Delayed: 0.077).

**Figure 19:**
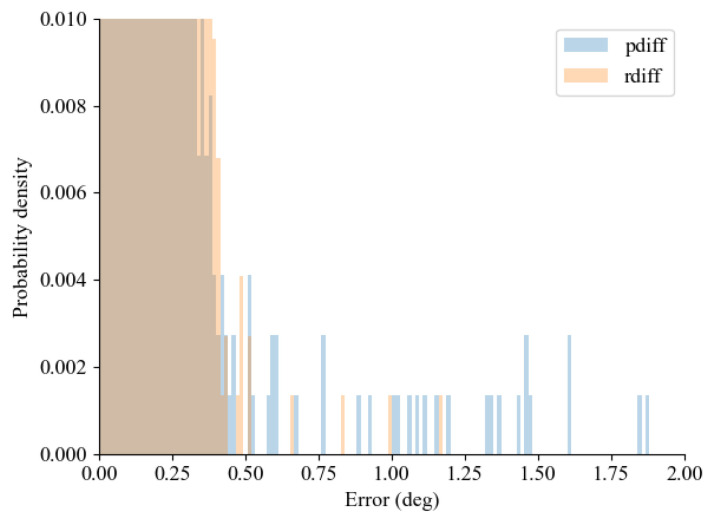
Probability density histogram showing that the predictor-based approach contains more erroneous samples.

**Figure 20:**
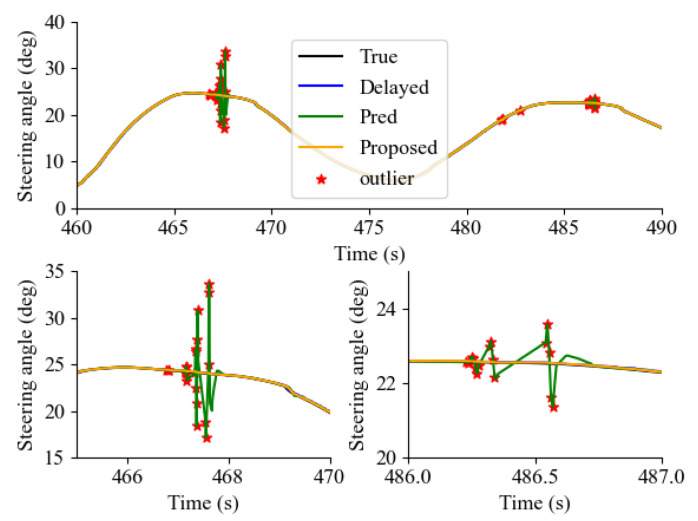
Example scene of outlier compensation predictor-based framework and original predictor-based framework.

As previously indicated, Figure 19 demonstrates that the predictor-based approach exhibits a higher count of erroneous samples in comparison to delayed signal samples. This observation implies that the predictor-based approach may be vulnerable to extreme scenarios where communication delay outliers coexist. Therefore, by using (26), the effect of communication outliers was suppressed, as shown in Figure 20. In Figure 20, the occurrence of a communication outlier is marked with an asterisk (*), illustrating the behavior of the original predictor-based approach under such circumstances. Specifically, when encountering communication delay outliers, it was observed that the sudden increase in communication delay resulted in an over/underestimation of the derivative of the predicted states. On the other hand, employing the proposed method within the outlier compensation predictor-based framework led to a notable decrease in the effect of the outlier because the derivative of the predicted state was not updated.

**Figure 21:**
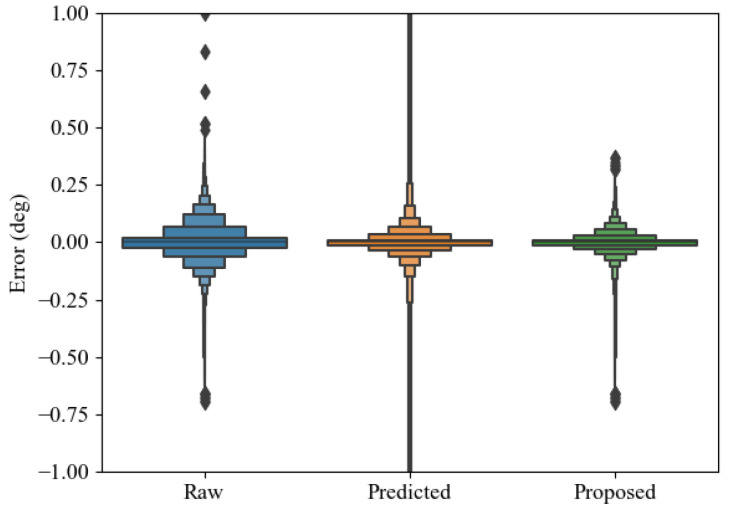
Comparison of estimated error between each method and non-delayed signal samples. The diamond markers represent the estimated outlier among the groups.

Figure 21 shows a comparison of the estimated error between each method and non-delayed signal samples. As a result of implementing the proposed method to reduce the effect of the communication delay outlier, the mean error and the deviations decreased, as shown in Table 1.

### 4.5. Monte Carlo Simulation Analysis against Different Communication Delays

**Figure 22:**
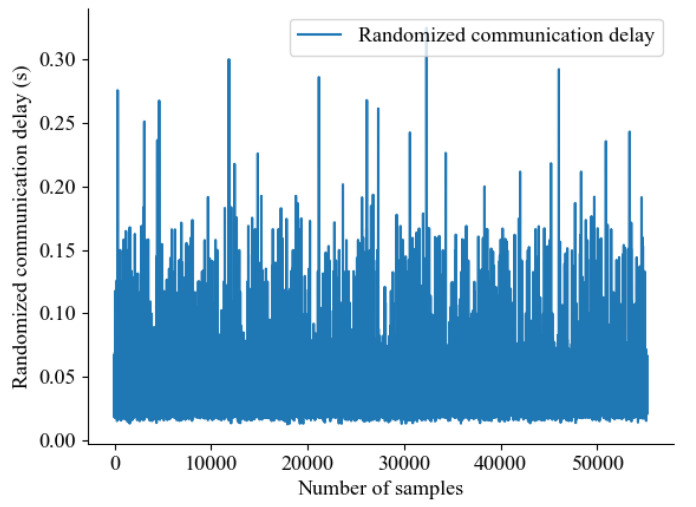
Randomized communication delay samples based on the measured communication delay samples.

Figure 22 shows randomized communication delay samples based on the measured communication delay samples. Here, we used mean square error as a comparison criterion as follows:

(27) $$\mathrm{RMSE}=\frac{1}{N}\sum_{k=1}^{N}\sqrt{\frac{1}{L}\sum_{i=1}^{L}{\left(\theta_{k}^{i}-{\hat{\theta }}_{k}^{i}\right)}^{2}},$$

where *L* is a sequence of trials for the simulation, *N* is the number of samples, and $\hat{\theta }$ is the compensated outlier sample. As for the comparison, the following parameters were used in the simulation ($L:100,N=N_{s}$, where $N_{s}$ refers to the number of samples). In addition, for different communication delay conditions, artificially generated delay samples ${\hat{T}}_{c}$ were used as follows:

(28) $${\hat{T}}_{c}=\sigma T_{c}+\mu ,$$

where σ is a scaler for the deviations, and μ is a scaler for the average communication delay.

**Figure 23:**
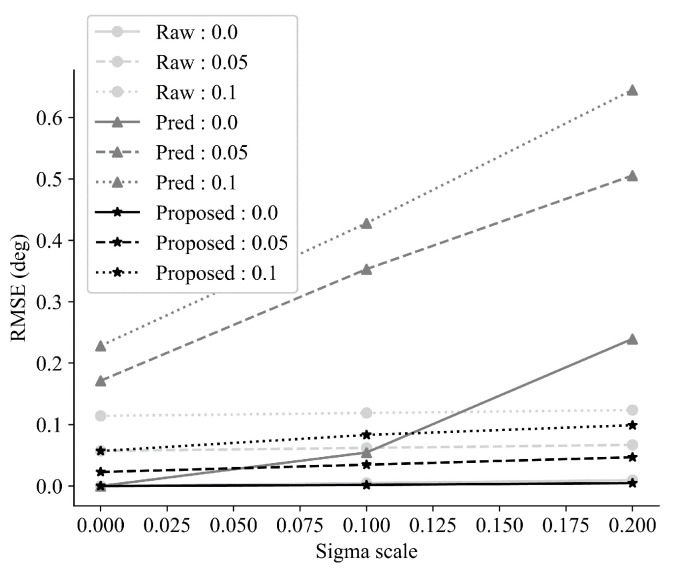
Comparison of RMSE for each method using the Monte Carlo simulation with differing μ (0 s, 0.1 s, 0.2 s) and σ (0, 0.05, 0.10).

Figure 23 shows a comparison of the RMSE for each method, including the proposed approach. From the result, it can be seen that our proposed method can resist the communication delay outlier compared to the predictor-based approach in terms of RMSE comparison.

The results underscore the significant ramifications of communication delay outliers in teleoperation, particularly in critical situations like emergencies and collision avoidance scenarios. These outliers can exacerbate challenges in maintaining situational awareness, potentially leading to confusion or misinterpretation of the remote environment, thus compromising decision-making accuracy.

To effectively address these implications, robust strategies for detecting and mitigating communication delay outliers in teleoperation systems are imperative. We advocate for the adoption of the proposed method for detecting communication delay outliers, which can complement existing approaches aimed at bolstering the resilience of teleoperation control signals.

This recommendation extends beyond vehicular applications to encompass various frameworks utilizing teleoperation, such as surgical robots, unmanned ground vehicles (UGVs), and drones. By implementing such strategies across these diverse domains, we can enhance the reliability and safety of teleoperation systems, ultimately benefiting a wide range of applications and industries.

## 5. Conclusions

From the perspective of vehicle teleoperation, accurate state estimation accounting for communication delays is crucial. To the best of the author’s knowledge, there has been limited exploration of communication delay estimation using a stochastic approach.

This study introduces a novel method for distinguishing between outliers in communication delays (ODs) and nominal passive delays (PDs) using stochastic criteria. By conducting teleoperation experiments, one-way communication delays were gathered and analyzed, with PDs and ODs estimated at 98.67% and 1.33%, respectively. The findings demonstrate the effectiveness of the proposed approach under both stable and dynamic communication conditions, with an estimated coverage interval of 5.16 σ = 25 ms to 100 ms.

Notably, the proposed outlier detection algorithm enhances performance by compensating for detected outliers within a predictor-based framework. However, it is important to note that the duration of time synchronization and related parameters must be thoroughly investigated to avoid compromising estimation performance.

Future research should focus on implementing a delay compensation strategy using the proposed method, offering promising avenues for enhancing vehicle teleoperation, particularly in scenarios where real-time responsiveness is critical. Furthermore, exploring the applicability of this approach in other domains, such as remote control of robots or drones, holds significant potential for advancement.

## Figures and Tables

**Table 1:** Comparison result of mean error and standard deviation between raw samples, predictor-based framework, and proposed outlier compensated predictor.

Index	Raw Samples	Predictor	Outlier Compensated Predictor
Mean error (deg)	$3.58\times 10^{-4}$	$9.58\times 10^{-3}$	$1.08\times 10^{-4}$
Standard deviation (deg)	$7.7\times 10^{-2}$	$3.54\times 10^{0}$	$4.72\times 10^{-2}$

## Data Availability

Data are contained within the article.
